# Editorial: Outsmarting the Host: How Bacterial Pathogens Modulate Immune Responses in the Lung

**DOI:** 10.3389/fimmu.2020.629491

**Published:** 2021-01-18

**Authors:** Michael T. Borchers, Gee W. Lau, Charles S. Dela Cruz

**Affiliations:** ^1^ Division of Pulmonary, Critical Care and Sleep Medicine, University of Cincinnati College of Medicine, Cincinnati, OH, United States; ^2^ Department of Veterans Affairs, Cincinnati VA Hospital, Cincinnati, OH, United States; ^3^ Department of Pathobiology, University of Illinois at Urbana-Champaign, Urbana, IL, United States; ^4^ Department of Internal Medicine, Center for Pulmonary Infection Research and Treatment, Yale University, New Haven, CT, United States

**Keywords:** bacteria, pathogen, host response, lung, immune response

Every day, thousands of bacteria gain access into the respiratory epithelium. This contact initiates complex interactions between the lung and the microbes. Host environments, including temperature, pH, ionic strength, mucus viscosity, and pulmonary surfactant, prompt bacteria to modulate genes required for invasion, evasion, and proliferation. The lung evokes both innate and acquired immune defenses to counter potentially pathogenic bacterial infections. Taking advantage of the compromised lung immunity caused by primary viral infection, external exposures, impaired mucus clearance mechanisms, ventilator-associated injuries, and other factors, bacterial pathogens and opportunistic commensals establish infection by deploying multi-pronged strategies that involve the bacterial capsule, cell wall, surface appendages, biofilms, secreted and injected virulence determinants. The interactions between bacteria and host result in varied clinical outcomes ranging from full recovery, acute necrotizing pneumonia, and pneumonia-derived sepsis and multiorgan dysfunction, to the persistent biofilm-dominated infection in chronically-diseased airways. Understanding the complex interactions between the respiratory epithelium, host immunity and bacterial pathogens may allow more effective therapeutic strategies against pneumonia.

This collection of original articles and reviews highlights current knowledge and research trends of bacterial pathogenesis in the context of pulmonary immunity and function, and updates ongoing and future antibacterial strategies. The collection of articles presented include topics such as; 1) Bacterial virulence determinants that mediate evasion, subversion, and modulation of innate and adaptive immunity, 2) Host factors involved in pathogen recognition and determination of the success and failure of lung infection, such as innate immune receptors and downstream signaling mechanisms, 3) The influence of antibacterials, antibacterial resistance, and phage therapy in modulating lung immunity, and 4) Non-antibiotic based immuno-modulating therapies.

Tuberculosis (TB) pathogenesis is characterized by inadequate immune activation and delayed T cell responses, which has driven recent immunotherapeutic efforts against *Mycobacteria tuberculosis* (MTB). Lim et al. investigated the immunostimulatory properties of bacterial ghosts (BG) as a novel approach to potentiate host immunity against mycobacterial infection. BG are intact cytoplasm-free *Escherichia coli* envelopes, previously developed as bacterial vaccines and adjuvant/delivery systems in cancer immunotherapy but have yet to be exploited in infectious diseases. The authors showed that BG modulate dendritic cell populations important for the development of T cells. BG also induced macrophage activation, which was associated with enhanced nitric oxide production, a key anti-mycobacterial weapon. Treatment with BG but not LPS reduced the mycobacterial burden in infected mice, correlating with increased influx of effector immune cells and elevated cytokines in the lungs. Importantly, enhanced MTB killing was seen in mice co-administered BG and second-line TB drugs (bedaquiline and delamanid), highlighting the role of BG as potential adjunct immunostimulators in mycobacterial treatment.

Alveolar macrophages play a key role in the development of a robust adaptive immunity against MTB. However, macrophage function is frequently hampered by the potent immune suppressor interleukin (IL)-10 where its level often correlates with disease pathogenesis and pathogen persistence. Bouzeyen et al. investigated the role of the forkhead box O-3 (FOXO3) transcription factor in mycobacterial-induced secretion of IL-10. They found that the FOXO3 axis regulated IL-10 expression in human macrophages. Genetic knockdown of FOXO3 resulted in increased IL-10 production in bacillus Calmette-Guérin (BCG)-infected macrophages. Co-culture studies showed human lymphocytes with FOXO3-transfected macrophages loaded with mycobacterial antigens had decreased expression of Th1/Th17 specific markers and increased expression of IL-10. These studies highlight FOXO3 as a potential target for host-directed therapies in TB.

Global control of TB remains elusive, and the BCG vaccine has proven insufficient for reversing this epidemic. Some have suggested that mass BCG vaccination might have affected MTB population structure. This is reflected in the prevalence of strains with higher ability to circumvent BCG-induced immunity such as the recent Beijing genotype. Zatarain-Barron et al. explored the interaction between MTB Beijing-genotype strains and BCG-vaccinated hosts. Using a mouse model of progressive pulmonary tuberculosis, they vaccinated mice with two sub-strains of BCG (BCG-Phipps and BCG-Vietnam). Following vaccination, the mice were infected with one of three selected MTB strains (two Beijing-family clinical isolate strains 46 and 48 and laboratory strain H37Rv). The authors showed that the lungs of the BCG vaccinated hosts select for MTB strains with higher-virulence, specifically Beijing genotype strains 46 and 48, through mutations in several virulence factors. These mutant MTB strains bacteria had an increased ability for immune evasion using and replicated more efficiently compared to bacteria collected from unvaccinated hosts or to the original-stock strain. These results highlight novel host-pathogen interactions induced by exposure of MTB to BCG vaccinated hosts.

*Streptococcus pneumoniae* is a major cause of respiratory infections in both children and the elderly worldwide. Serotype replacement is frequent after the introduction of conjugated vaccines, with emerging serotypes 22F and 33F as frequent non-PCV13 serotypes. Characterization of mechanisms of host immune evasion by serotypes 22F and 33F is important given their inclusion in future conjugated vaccines. Sempere et al. evaluated the influence of capsule polysaccharide in biofilm formation and immune evasion in multiple clinical isolates. They demonstrated that pediatric isolates of serotypes 22F and 33F formed better biofilms than adult isolates, possibly providing an advantage in colonizing children’s nasopharynx and may be important in the carriage and dissemination to the elderly. The biofilm phenotype was greatest in clinical isolates from blood compared to other sites. Serotype 22F was more resistant than 33F to phagocytic killing and more lethal in a mouse sepsis model. The increased ability of serotype 22F to avoid the host immune response might explain the emergence of this serotype in recent years.

Cyclic di-AMP (c-di-AMP) is an important signaling molecule for pneumococci. As a uniquely prokaryotic product, it can be recognized by mammalian cells as a danger signal that triggers innate immunity. Wooten et al. used pneumococcal mutants with defective c-di-AMP catabolism to examine its role in mediating host responses. Pneumococci deficient in phosphodiesterase 2 stimulated a rapid and exaggerated interferon β (IFNβ) response in macrophages when compared to that induced by wild type or mutant deficient in phosphodiesterase 1. Macrophage hyperactivation by Pde2-deficient pneumococci led to rapid cell death. Additionally, STING and cGAS were essential for the excessive IFNβ induction, bacterial phagocytosis, and IRF3/IRF7 activation. These findings suggest that bacterial c-di-AMP is pivotal for innate immune response against pneumococci.

*Francisella tularensis* (Ft) is a highly virulent intracellular Gram-negative bacteria that causes pneumonic tularemia, characterized by severe alveolitis marked by excessive neutrophilia. Ft suppresses neutrophils by impairing their respiratory burst and phagocytic activity. However, the fate of the massive numbers of neutrophils recruited to the infection site is unclear. Pulavendran et al. showed that Ft infection resulted in prominent induction of neutrophil extracellular traps (NETs) within infected lungs of mice, domestic cats, and rabbits. Infection with a live attenuated strain of Ft resulted in increased NETosis, as noted by increased lung myeloperoxidase (MPO) and peptidylarginine deiminase 4. They also found that NETs exacerbated epithelial damage, and that targeting NETosis, including MPO, may offer novel therapeutic interventions in pulmonary tularemia.

*Staphylococcus aureus*, an important and prevalent pathogen in the airways of cystic fibrosis (CF) patients, also triggers a strong neutrophilic response. Herzog et al. hypothesized that *S. aureus* adapts to CF airways by escaping from NET-mediated killing *via* an increase in nuclease activity. They examined CF sputum samples and found that chronic *S. aureus* infection was associated with extracellular DNA structures in the CF sputa. NET-mediated killing was significantly higher in *S. aureus* isolates with low nuclease activity compared to those with high nuclease activity. Importantly, overexpression of nuclease Nuc1 in clinical isolates with low nuclease activity conferred protection against NET-mediated killing. The authors also reported that nuclease expression was high in CF sputa, highlighting the potential of targeting nuclease expression in order to disrupt the success of *S. aureus* adaptation to chronic infection in CF airways.

The Src homology 2-containing inositol 5-phosphatase (SHIP-1) is an inositol phosphatase that modulates a variety of cellular processes by hydrolyzing phosphatidylinositol 3-kinase (PI3K) products and negatively regulating protein kinase B (Akt) activity. However, the role of SHIP-1 in bacterial-induced sepsis is largely unknown. Qin et al. showed that SHIP-1 regulates inflammatory responses during *Pseudomonas aeruginosa* infection. Infected SHIP-1^−/−^ mice had decreased survival rates and increased proinflammatory responses, owing to elevated expression of PI3K over that seen in wild-type mice. Inhibition of SHIP-1 resulted in lipid raft aggregates, aggravated oxidative damage, and bacterial accumulation in macrophages after infection by *P. aeruginosa* strain PAO1. SHIP-1 deficiency augmented phosphorylation of PI3K in a manner that altered the differentiation of macrophages. These findings revealed a previously unrecognized role of SHIP-1 in inflammatory and macrophage homeostasis during *P. aeruginosa* infection.

The airway epithelium and underlying innate immune cells comprise the first line of host defense in the lung. They recognize pathogen-associated molecular patterns using membrane-bound receptors, as well as cytosolic receptors such as inflammasomes involving specific caspases, IL-1β, and IL-18, as part of lytic cell death termed pyroptosis. This includes the caspase-11 inflammasome important in defense against Gram-negative pathogens. However, pathogens can employ evasion strategies to minimize or evade host caspase-11 detection. Oh et al. presented a comprehensive overview of caspase-11 in sensing cytosolic lipopolysaccharide (LPS), a bacterial cell wall component, as well as the strategies pathogens use to evade caspase-11.

A number of pulmonary pathogens have the ability to evade normal mucosal defenses to establish acute infection and then adapt to cause chronic pneumonias. Riquelme et al. examined how the inherent metabolic flexibility of *P. aeruginosa* and *S. aureus* drives their success in chronic lung diseases such as CF and chronic obstructive pulmonary disease (COPD). To establish infection, these bacteria expressed virulence factors that facilitate colonization. However, much less is known about the adaptive changes *in vivo* that allow *P. aeruginosa* and *S. aureus* to evade immune clearance. These colonizers proliferated and generated small colony and mucoid variants that were optimized for long term infection. Such host-adapted strains evolved in response to selective pressure such as antibiotics and the recruitment of phagocytes at sites of infection and their release of signaling metabolites (e.g., succinate). These metabolites could spur bacterial growth but also cause oxidant stress. The serial isolation of clonally related strains from CF patients identified bacterial metabolic pathways that are altered under this immune pressure, including the antioxidant glyoxylate and pentose phosphate pathways, which contribute to biofilm formation. The authors concluded that host immune signaling metabolites drive bacterial adaptation and promotes their persistence in the airways.

Lung epithelial cells have multiple direct antibacterial mechanisms that include mucus secretion, mucociliary clearance, antimicrobial peptide production, and phagocytosis. Secretion of cytokines to recruit immune cells and potentiate their antimicrobial activities also contributes to bacterial clearance. Successful pathogens outsmart epithelial resistance to replicate in sufficient numbers and establish infections in the airway or lung epithelial surfaces. Sharma et al. discussed the many roles of respiratory epithelial cells versus immune cells such as macrophages and neutrophils in bacterial clearance. The authors summarized data that establish the importance of epithelial host defense against invading respiratory bacterial pathogens and discuss how these pathogens outsmart these immune mechanisms to successfully establish infection. Additionally, they discussed how to boost epithelial immunity to improve outcomes in bacterial lung infections.

Under pathological conditions such as chronic lung inflammation, bacteria employ various mechanisms from structural changes to protease secretion to evade the host immune responses, creating a niche permitting commensal bacteria to thrive. Therefore, understanding the mechanisms by which pathogenic bacteria survive in the host tissues may offer new strategies to overcome persistent bacterial infections. Mues and Chu reviewed how certain pathological conditions could impair normal defense barriers leading to bacterial survival and persistence. The authors described the complex interactions between pathogenic bacteria and immune responses in several major chronic airway inflammatory diseases such as asthma and COPD.

Pf bacteriophage are temperate phages that infect *P. aeruginosa*. Pf and other temperate phages have evolved complex, mutualistic relationships with their bacterial hosts. Secor et al. reviewed the biology of these phages and their contributions to bacterial pathogenesis and clinical disease. They discussed the structure, genetics, and epidemiology of Pf phages, as well as their role in *P. aeruginosa* biofilm formation, antibiotic resistance, and motility. Additionally, they discussed experimental evidence that Pf phages suppress mammalian immunity at sites of bacterial infection pointing to recent studies implicating a role of these phages in chronic *P. aeruginosa* infections in CF and other clinical settings.

Forkhead box (FOX) proteins are transcriptional factors that regulate various cellular processes. Choi et al. provided an overview of crucial roles played by FOXA2 during lung morphogenesis, surfactant protein production, goblet cell differentiation and mucin expression, with an emphasis on airway mucus homeostasis. In healthy airways, FOXA2 exerts tight control over goblet cell development and mucin biosynthesis. However, in diseased airways, microbial infections and proinflammatory responses deplete FOXA2 expression, resulting in uncontrolled goblet cell hyperplasia and metaplasia, mucus hypersecretion, and impaired mucociliary clearance of pathogens. The accumulated mucus creates a niche environment for persistent microbial colonization and infection, leading to acute exacerbation and deterioration of pulmonary function. The authors discussed how FOXA2 is inhibited by antagonistic signaling pathways as well as through posttranslational modifications induced by microbial infections. They proposed that increased understanding of FOXA2 biology may lead to novel therapeutics that preserve the protein’s function, which in turn, improve the mucus status and mucociliary clearance of pathogens.

Alveolar and interstitial macrophages infected with MTB frequently differentiate into lipid-laden foamy macrophages, a hallmark of TB granuloma. Shim et al. discussed the current understanding of the pathogenic alterations in both alveolar and interstitial macrophages in the regulation of MTB -induced immune responses. Metabolic reprogramming of lipid-laden foamy macrophages following MTB infection or exposure to its virulence factors was also summarized. They also reviewed the therapeutic interventions targeting immune responses and metabolic pathways in pre-clinical and clinical studies, highlighting the therapeutic potential against TB.

Both hosts and pathogens have evolved mechanisms to regulate the process of host cell death during lung infection. The host aims to rapidly induce an inflammatory response at the site of infection, promote pathogen clearance, resolve inflammation, and return to tissue homeostasis. The appropriate modulation of cell death in respiratory epithelial cells and pulmonary immune cells is central in the execution of all of these processes. Cell death can be inflammatory or anti-inflammatory, depending on the regulated cell death (RCD) modality triggered and the infection context. Diverse bacterial pathogens have also evolved many means to manipulate host cell death to increase pathogen survival and spread. FitzGerald et al. outlined several current lines of research characterizing bacterial pathogen manipulation of cell death pathways in lung cells. They postulated that understanding the dynamics of these interactions and RCD manipulation by intracellular and extracellular bacteria may lead to novel therapeutic approaches for the treatment of intractable respiratory infections.

Because of increased antibiotic resistance, chronic bacterial lung infections represent a growing threat to human life. Many bacteria directly target inflammatory cells and cytokines to impair host responses. However, bacteria also have the capacity to promote anti-inflammatory responses in the lung to counter local pro-inflammatory responses critical for bacterial elimination. For example, regulatory T cells and myeloid-derived suppressor cells are often enhanced in number and activity to promote a permissive environment for chronic lung infection. Kelly and McLoughlin explored the anti-inflammatory component of the lung immune system that are targeted by bacteria, and discussed how bacterial-induced immunosuppression could be inhibited through the use of host-directed therapies to improve treatment options for chronic lung infections.

This Research Topic brings together an informative and updated collection of original research and literature reviews that better define the past and present problems associated with the complex interactions between bacterial pathogens and the host pulmonary immune response. These reports provide a robust background and exciting original findings that we hope will spur new ideas and strategies to enhance host immunity to develop more effective therapeutic strategies against bacterial pneumonias.

## Author Contributions

All authors contributed equally to the editorial. All authors contributed to the article and approved the submitted version.

## Funding

This work was funded in part by the support from the NIH (HL119538 to MTB, AI080710 and HL142626 to GWL; and HL126094 to CDC) and the Veteran’s Administration (I01BX002347 to MTB and I01BX004661 to CDC).

## Conflict of Interest

The authors declare that the research was conducted in the absence of any commercial or financial relationships that could be construed as a potential conflict of interest.

